# Far-lateral craniotomy for obliteration of foramen magnum dural arteriovenous fistula

**DOI:** 10.3171/2025.7.FOCVID2579

**Published:** 2025-10-01

**Authors:** Michael G. Brandel, Jeffrey A. Steinberg

**Affiliations:** Department of Neurosurgery, University of California, San Diego, California

**Keywords:** infratentorial, vascular malformation, angiogram, indocyanine green, cervicomedullary junction, treatment

## Abstract

A 63-year-old woman with a history of stroke underwent further workup. MRI revealed a region of asymmetrical ASL hyperintensity near the right hypoglossal canal. Angiogram revealed a left foramen magnum region dural arteriovenous fistula supplied by a left V4 vertebral artery branch, with early draining veins into the skull base and left sigmoid sinus. Attempts were made to catheterize the fistula, but it was not amenable to endovascular intervention. A far-lateral craniotomy with C1 hemilaminectomy was performed, with obliteration confirmed on postoperative DSA. At 2-year follow-up, the patient remained at her neurological baseline with no evidence of recurrence.

The video can be found here: https://stream.cadmore.media/r10.3171/2025.7.FOCVID2579

## Transcript

In this video, we will demonstrate a far-lateral craniotomy for obliteration of foramen magnum dural AV fistula.

### 0:29 Clinical Presentation and Exam.

This is a 63-year-old female with a history of stroke. She was incidentally found to have a possible dural AV fistula at the foramen magnum.

She had headaches but no focal neurological deficits. MRI brain demonstrated increased ASL signal within the region of the right hypoglossal canal and plexus. We recommended a digital subtraction angiogram for further evaluation and potential treatment options.

### 0:54 Preoperative Imaging.

On the MRI, there is elevated ASL signal, seen by the white arrow. This is eccentric to the right in the premedullary area and the hypoglossal canal. On the center image, the right vertebral artery is demonstrated by the arrow.

Just midline to this, there is a prominent tortuous vessel concerning for fistula site. On the far-right image, there is enhancement in the right hypoglossal canal. Overall, these findings were concerning for a foramen magnum dural AV fistula.

However, the gold-standard diagnostic cerebral angiogram was recommended for further characterization. An AP view of the left vertebral artery is demonstrated. There’s a prominent tortuous vessel coming off the V4 segment with filling into an early venous drainage system going into the right hypoglossal canal and up toward the left transverse sigmoid junction.

After the angiogram was completed, a discussion was held with the patient. The first decision point was whether to treat the fistula or not. Given the venous reflux, it was felt that treatment was indicated to prevent hemorrhage or other complications.

During the catheter angiogram, microcatheterization of the feeding vessel off the vertebral artery had been attempted. However, given the small tortuous nature, it was unsuccessful. Given this finding, endovascular options were felt to be limited for embolization.

Additionally, embolization reflux would have been very limited. Given these findings, a far-lateral craniotomy was recommended for obliteration of the fistula. A 3D reconstruction was completed from a CTA demonstrating the left V4 segment of the vertebral artery.

Originating at this location is the tortuous vessel seen in this image. This was felt to be the fistulous connection point, and the 3D reconstruction was utilized for preoperative planning. For the surgery, general anesthesia with neuromonitoring was planned.

The patient was positioned prone in the Mayfield head holder. She had a thin body habitus and thus it was felt we could get eccentric to the left in this position. Surgical adjuncts included Brainlab neuronavigation, micro-Doppler, indocyanine green, and aneurysm titanium clips.

Although we often perform intraoperative angiograms to ensure fistula obliteration, this patient had a history of stroke with significant atherosclerotic disease within her cranial and cervical vasculature. Completing an intraoperative angiogram was felt to be too high-risk for complications.

### 3:31 Initial Exposure After Completion of C1 Laminectomy.

In this view, we are eccentric to the left at the cervicomedullary junction.

To the left of the screen is the suboccipital dura. At the right inferior portion of the screen is the remaining C1 lamina after completing a C1 laminectomy, eccentric to the left.

Here we are using a round diamond drill to drill down the occipital condyle to allow opening of the dura as far lateral as possible. A Doppler is utilized to localize the vertebral artery. This is the view after opening up the dura.

We then open up the arachnoid with sharp microdissection. At this point, we can see the underside of the cerebellum, and now we are mobilizing cranial nerve XI. The dentate ligament is visualized and cut for further visualization and mobilization.

### 4:22 Exposure of the Fistula After Dividing the Dentate Ligament.

At this point, prominent vasculature is seen running along the lateral aspect of the cervicomedullary junction in the vertebral artery. Microdissection is utilized to visualize these vessels and free them from the tethering arachnoid. Here a prominent vessel is seen.

Micro-Doppler is utilized to demonstrate it is arterialized. Here is the origin of the vertebral artery intradurally. We continue to free up these vessels to understand the architecture.

At this point, we begin to visualize vessels originating off the vertebral artery and likely the source of the fistula. We continue to dissect these free and utilize micro-Doppler. These vessels are arterialized.

Again, we free up all vessels that may be related to the fistula.

### 5:18 First Indocyanine Green Injection Demonstrating the Fistula.

At this point, we complete an indocyanine green injection demonstrating the arterialized hypervascularity along the left cervicomedullary junction. We place some mini–aneurysm clips on these vessels coming off the vertebral artery.

We continuously are doing our neuromonitoring. No changes are encountered with neuromonitoring after placing these clips and waiting 5 minutes. We utilize the Doppler to check for decreased arterialized signal.

### 5:52 Second Indocyanine Green Injection Demonstrating a Small Amount of Residual Fistula.

At this point, we again complete an indocyanine green demonstrating decreased vascularity but still some prominent vessels. There is decreased Doppler signal in these vessels. However, to ensure complete obliteration, cautery is utilized to take down the fistula.

Careful dissection and bipolar of these vessels is completed. At this point, we are satisfied that the fistula is obliterated.

### 6:21 Final Indocyanine Green Injection Demonstrating Fistula Obliteration.

A final ICG demonstrates normal filling of the spinal cord with no early venous drainage.

Postoperative imaging is demonstrated. On the left of the screen is an axial CT scan demonstrating the aneurysm clips at the origin of the fistula off the vertebral artery. A 3D reconstruction of our CT scan demonstrates the far-lateral craniotomy eccentric to the left.

And on the right of the screen is the ASL postoperatively demonstrating no residual high flow associated with the fistula. Postoperative angiogram of the left vertebral artery demonstrates complete obliteration of the fistula.

### 7:05 Postoperative Outcome.

The patient had an uneventful postoperative course with no complications.

She had some neck pain requiring pain management and was discharged on postoperative day 9. At 2-year follow-up, she was at her neurological baseline with no focal deficits. Surveillance MRI demonstrates no evidence of increased ASL signal and no evidence of recurrence.

In conclusion, dural AVFs of the foramen magnum are rare. Optimal treatment strategies are not well defined.^1–5^ Endovascular treatment can be considered.

However, feeding vessels to the fistula may be difficult to catheterize and may supply the cervical medullary region. An infratentorial craniotomy and often a far-lateral craniotomy may be utilized to obliterate the fistula via an open approach. We perform preoperative and postoperative catheter angiogram to ensure complete obliteration has been accomplished.

When feasible, intraoperative angiogram can be considered.

## Disclosures

The authors report no conflict of interest concerning the materials or methods used in this study or the findings specified in this publication.

## Author Contributions

Primary surgeon: Steinberg. Editing and drafting the video and abstract: both authors. Critically revising the work: both authors. Reviewed submitted version of the work: both authors. Approved the final version of the work on behalf of both authors: Steinberg. Supervision: Steinberg.

## Supplemental Information

### Patient Informed Consent

The necessary patient informed consent was obtained in this study.
